# Investigating Detectability of Infrared Radiation Based on Image Evaluation for Engine Flame

**DOI:** 10.3390/e21100946

**Published:** 2019-09-27

**Authors:** Xia Li, Jun Wang, Meihui Li, Zhenming Peng, Xingrun Liu

**Affiliations:** 1 The Science and Technology on Optical Radiation Laboratory, Beijing 100854, China; lixia207@sina.com (X.L.); wangjunt@vip.sina.com (J.W.); liuxr207@126.com (X.L.); 2 School of Information and Communication Engineering, University of Electronic Science and Technology of China, Chengdu 611731, China; meihuili@std.uestc.edu.cn; 3 Laboratory of Imaging Detection and Intelligent Perception, University of Electronic Science and Technology of China, Chengdu 610054, China

**Keywords:** atmosphere background, engine flame, infrared radiation, detectability, image quality evaluation

## Abstract

Aiming at the application requirements of infrared detection, the influence of earth background interference on plume radiation detection is investigated and discussed in this article. The infrared image of the earth’s atmospheric background radiation is simulated by the spectral correlation based on the conversion model of the surface radiation with different bands. The infrared radiation image of the jet flame and the background is generated by overlapping the infrared radiation of the engine flame and the background radiation according to the detection angle of view. Through the image quality evaluation model, the detectability of the flame is analyzed. The simulating results show that the comprehensive statistical features such as image information entropy, variance and signal-to-clutter ratio can be used to evaluate the detectability of the engine flame.

## 1. Introduction

The detectability investigation of infrared radiation gives very important guiding and reference for the performance evaluation and design of infrared detection system [1,2,3]. The detection of the rocket engine flame in flight state plays an important role in military affairs. Due to the complexity of the infrared imaging process, the rocket exhaust plume is presented as a small dim target in the image, which is hard to detect using existing techniques. Thus, many improvements have been made by researches in recent years, including shearlet features [4,5], high-order cumulant [6], local energy [7], non-convex optimization with regularization constraint [8,9,10,11,12,13].

The background of the flying rocket engine flame is mainly the earth’s atmosphere background and deep space background. Deep space can be equivalent to 4K cold background and the atmosphere has strong selective absorption of infrared radiation from the plume. The background radiation of the earth also interferes with the detection of plume radiation. Therefore, it is of great significance to study the coupled radiative transfer characteristics between the plume and the earth’s atmosphere. The jet flow field and its radiation characteristics are complex physical and chemical processes in many disciplines. The research involves the interaction and flow process between the jet and the accompanying flow, the secondary combustion of some components, the spectral characteristics of components and the radiation transfer process. Atmospheric radiation transfer involves scattering and absorption of molecules and aerosols, as well as radiation scattering of the sun and the moon. Since the last century, scholars at home and abroad have carried out relevant research and formed a series of special computing software. For example, Fluent, CFD and CFD++ are used to calculate the flow field of jet, LOWTRAN and MODTRAN are used to calculate the atmospheric radiation transmission. But usually the rocket engine works at a certain altitude. In order to achieve its detection and evaluation, it is necessary to consider the energy state after the coupling between the flame and the atmosphere and it is also related to the detection angle and altitude.

The research of flame radiation and flow field modeling has been published in many papers [1,2,3]. This paper focuses on analyzing the detectability of flame from the perspective of image by using the existing target and background data, and builds an image-based analysis model of detectability. First, we uses MODIS remote sensing data and MODTRAN calculation data to simulate the infrared image of the earth’s atmospheric background radiation through the spectral correlation based conversion model of the surface radiation band. Then, the infrared radiation image of target and background is generated by overlapping the infrared radiation of engine exhaust flame and background radiation according to the detection angle of view. Finally, the detectability of plume radiation is studied and analyzed by some image quality evaluation model.

## 2. Infrared Radiation Calculation

### 2.1. Earth Background

**Figure 1:**
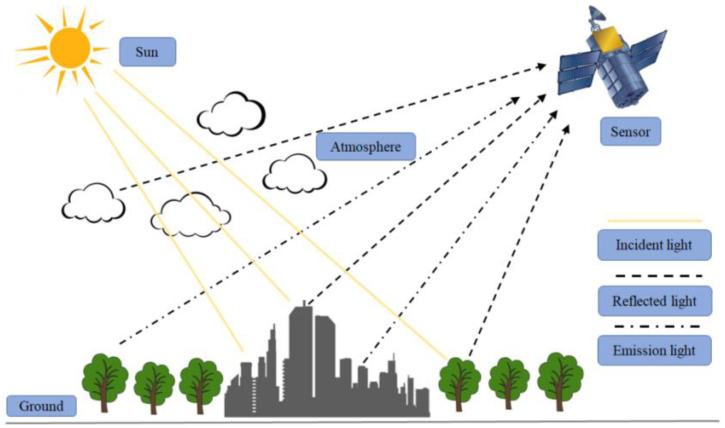
Diagram of the imaging process of a remote sensor.

In the process of sensor remote sensing imaging, the measured infrared image is the result of the interaction between surface, atmosphere and sensor. The imaging process is shown in Figure 1. The radiation received by the sensor is a comprehensive characterization of the solar radiation outside the atmosphere and the thermal radiation on the surface of the atmosphere. In this paper, the infrared radiation simulation method of the earth background in Reference [14], using MODIS remote sensing data and then, through adjacent channel band conversion [15], the infrared radiation image of the earth background is obtained.

### 2.2. Missile Flame

The line of sight (LOS) method combined with the single line group (SLG) model of single line group is used to solve the radiation transfer of jet flame. The transmission of *L* in radiation field is simplified to a problem of radiation transmission in multi-dimensional and multi-layered media. The flame region through which the line of sight passes is decomposed into *N* layers. The medium of each layer is considered homogeneous and isothermal. Considering the absorption and emission of each layer, the total infrared radiation intensity can be obtained by recursion step by step [16,17]. The formula is:

(1) $${\bar{I}}_{\Delta \eta }^{i}={\bar{I}}_{\Delta \eta }^{i-1}{\bar{\tau }}_{\Delta \eta }^{i}+{\bar{I}}_{b,\Delta \eta }^{i}\left(1-{\bar{\tau }}_{\Delta \eta }^{i}\right)$$

where ${\bar{I}}_{\Delta \eta }^{i}$ is average spectral radiation intensity, ${\bar{I}}_{b,\Delta \eta }^{i}$ is average spectral radiation intensity of blackbody, ${\bar{\tau }}_{\Delta \eta }^{i}$ is average transmittance.

## 3. Detectability Analysis of Flame Infrared Radiation

### 3.1. Generation of Infrared Radiation Data of Flame and Background

In order to analyze the detectability of engine exhaust plume, it is necessary to physically overlap the exhaust plume radiation with the background radiation. Firstly, the projection of the image plane is calculated according to the size of the jet and the spatial resolution of the sensor. Then the convolution calculation of the flame radiation spectrum observed by each pixel with the atmospheric transmittance spectrum and the sensor transmittance spectrum is carried out. Finally, the integration is carried out according to the band of the sensor. The energy distribution of the flame on the image plane of the sensor can be obtained. According to the maximum and minimum energy in the image, the gray level is linearly transformed and the energy infrared image is transformed into a gray level image. The determination of simulation band is based on the spectrum of flame and atmospheric transmission.

**Figure 2:**
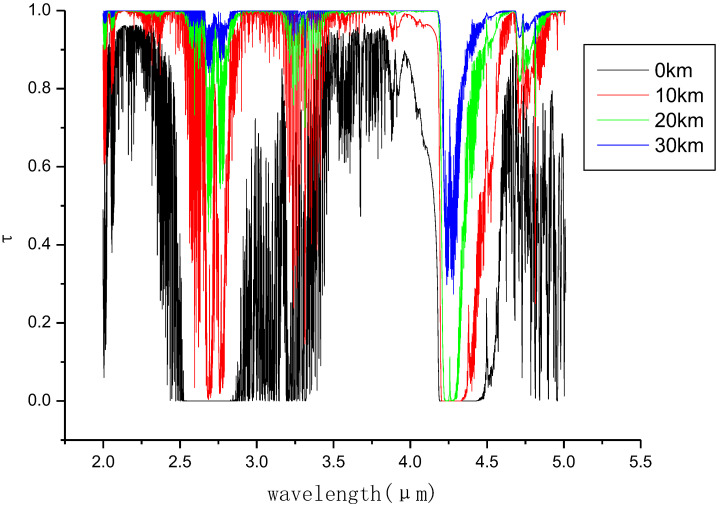
Atmospheric transmittance spectra from different altitudes to the outer atmosphere.

**Figure 3:**
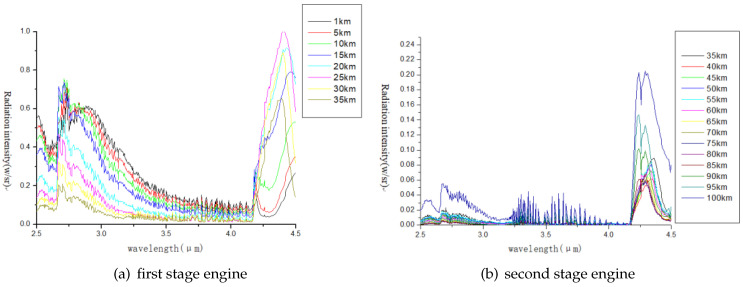
Normalization of engine flame radiation.

Figure 2 shows atmospheric transmittance spectra at different altitudes. We can see that 0.7–2.5 μm, 3–5 μm, 8–12 μm are three atmospheric windows, which are the range of electromagnetic wavelengths to which earth’s atmosphere is largely or partially transparent. Its low value zones (2.5–3.0 μm, 4.0–4.5 μm) indicate absorption bands. Figure 3 shows engine exhaust spectra at different flight altitudes.

From the comparison of Figure 2 and Figure 3, it can be seen that there are two radiation peaks at 2.5–3 μm and 4.0–4.5 μm. The two bands in the atmosphere are the absorption bands, which can effectively shield the earth background radiation to the sensor. Therefore, the simulation band is determined to be 2.5–3 μm and 4.0–4.5 μm. Four typical backgrounds are selected to simulate the cities, deserts, mountains and waters. The simulation time is daytime (solar zenith angle 15 degrees, relative azimuth 180 degrees), night and cloudless sky. Atmospheric model: Mid-latitude summer.

**Figure 4:**
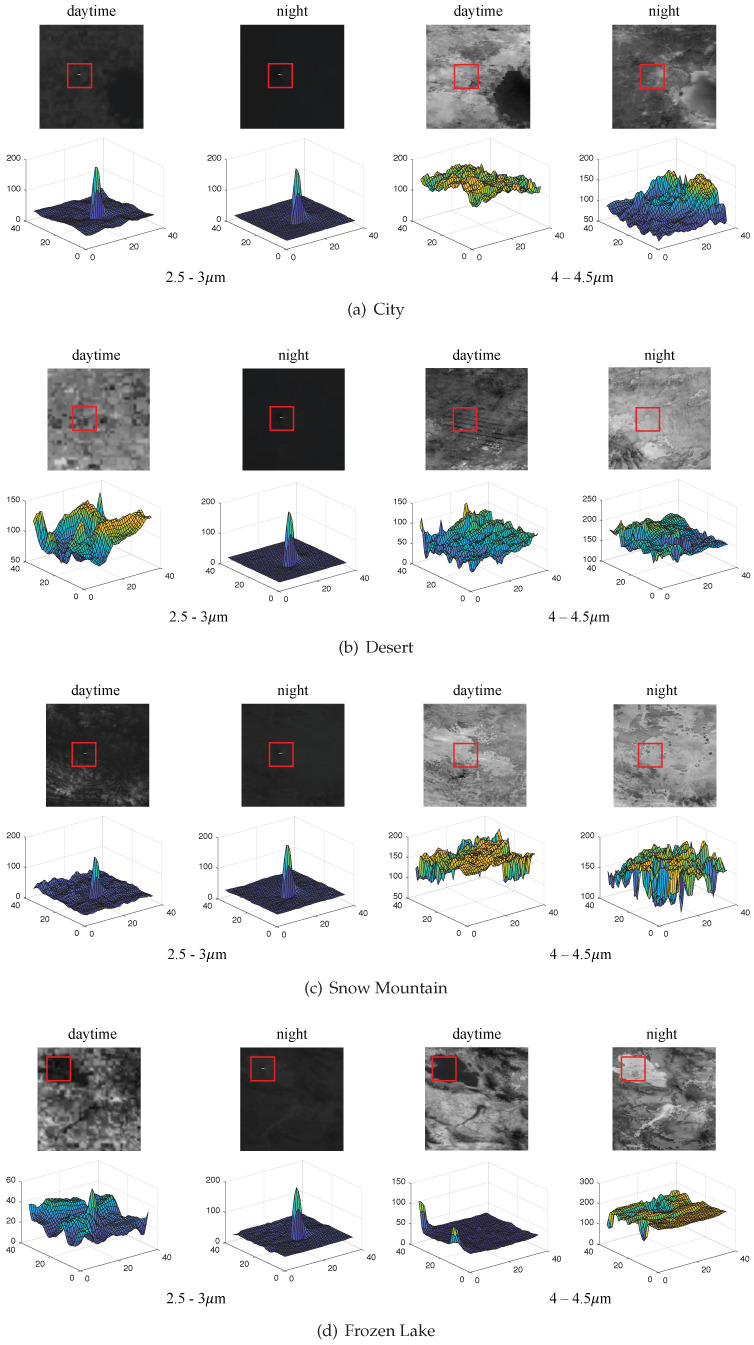
Infrared image of flame and background superimposed. The surface plots show the gray level of the target areas, which are labeled by the red bounding boxes in images.

Figure 4 shows the infrared radiation image of the jet and the earth’s atmosphere at the altitude of 30 km. For the sake of intuitive description, 3D surfaces of the local area (red rectangular box) including the flame are drawn, corresponding to the 2D graylevel image of the upper parts, respectively.

### 3.2. Detectability Analysis of Flame Radiation

Space sensors usually output infrared images. Generally speaking, there are several commonly used evaluation indicators to evaluate the imaging quality of an infrared image, such as peak signal-to-noise ratio (PSNR), signal-to-clutter ratio (SCR), information entropy (En), contrast (Contrast), structural similarity index measurement (SSIM), homogeneity (Hom), smoothness (Smo), variance (Var), skewness (Skew), kurtosis (Kur) and so forth. These indicators are defined as follows.

(1) Signal Effectiveness
  - Peak signal-to-noise ratio
    The calculation of the peak signal-to-noise ratio (PSNR) is based on the mean square error (MSE) and is defined by:
    (2) $$PSNR=10\log_{10}\left(\frac{MAX_{t}^{2}}{MSE}\right)$$
    where MSE is defined as:
    (3) $$MSE=\frac{1}{mn}\sum_{i=0}^{m-1}\sum_{j=0}^{n-1}{∥t\left(i,j\right)-b\left(i,j\right)∥}^{2}$$
    where *t* and *b* represent the target area and background area, respectively.
  - Signal-to-clutter ratio
    The signal-to-clutter ratio is defined as:
    (4) $$SCR=\frac{\left|\mu_{t}-\mu_{b}\right|}{\sigma_{b}}$$
    where $\mu_{t}$ is the average gray value of target pixels, $\mu_{b}$ is the average gray value of the background pixels, $\sigma_{b}$ is the standard deviation of the gray value of the background area.
  - Contrast
    The contrast describes the gradual change of image brightness [18]. A larger contrast value represents a richer gray level change of an image. The contrast is defined as:
    (5) $$C=\sum_{\delta }\delta {\left(i,j\right)}^{2}P_{\delta }\left(i,j\right)$$
    where $\delta \left(i,j\right)=∥i-j∥$ represents the gray difference between adjacent pixels. $P_{\delta }\left(i,j\right)$ represents the distribution probability of pixels whose gray difference is equal to δ.
(2) Statistical Characteristics
  - Variance
    Image variance is a measure of gray contrast and a measure of uniformity of sample distribution [19].
    (6) $$Var=\sum_{i=0}^{L-1}{\left(z_{i}-m\right)}^{2}p\left(z_{i}\right)$$
    where *z* is a random variable representing gray level, $p\left(z_{i}\right)$ is the corresponding histogram distribution, *m* is the mean of *z*.
  - Skewness
    The skewness of an image is defined by the third-order statistical moments [18]:
    (7) $$Skew=\sum_{i=0}^{L-1}{\left(z_{i}-m\right)}^{3}p\left(z_{i}\right)$$
  - Kurtosis
    The kurtosis of an image is defined by the fouth-order statistical moments [18]:
    (8) $$Kur=\sum_{i=0}^{L-1}{\left(z_{i}-m\right)}^{4}p\left(z_{i}\right)$$
(3) Texture
  - Homogeneity
    The homogeneity describes the variance of pixels within a region. It is defined as follows [18]:
    (9) $$Hom=\sum_{i=0}^{L-1}p^{2}\left(z_{i}\right)$$
    where *z* is a random variable representing gray level, $p\left(z_{i}\right)$ is histogram distribution, *L* is the number of different gray levels.
  - Smoothness
    The smoothness of an image is defined by the second-order statistical moments [18]:
    (10) $$Smo=\sum_{i=0}^{L-1}{\left(z_{i}-m\right)}^{2}p\left(z_{i}\right)$$
    where *m* is the average gray value of an image.
(4) Information
  The image entropy is the average number of bits per pixel in the gray level set of the image. The greater the image entropy, the more uniform the gray distribution of the image. The definition of image information entropy is as follows [20,21,22,23,24]:
  (11) $$En\left(z\right)=-\sum_{i=0}^{L-1}p\left(z_{i}\right)\log_{2}p\left(z_{i}\right)$$
  where *z* is a random variable representing gray level, $p\left(z_{i}\right)$ is histogram distribution, *L* is the number of different gray levels.
(5) Structural similarity
  The structural similarity index measurement(SSIM)is designed to improve on traditional methods such as PSNR and mean squared error(MSE) and is based on the image light, contrast and structure and is defined as [25]:
  (12) $$\begin{array}{c} l\left(T,B\right)=\frac{2\mu_{t}\mu_{b}+C_{1}}{\mu_{t}^{2}+\mu_{b}^{2}+C_{1}}\\ c\left(T,B\right)=\frac{2\sigma_{t}\sigma_{b}+C_{2}}{\sigma_{t}^{2}+\sigma_{b}^{2}+C_{2}}\\ s\left(T,YB\right)=\frac{\sigma_{tb}+C_{3}}{\sigma_{t}+\sigma_{b}+C_{3}}\\ SSIM\left(T,B\right)=l\left(T,B\right)\times c\left(T,B\right)\times s\left(T,B\right) \end{array}$$
  where $\mu_{t}$ is the average gray value of target pixels, $\mu_{b}$ is the average gray value of the background pixels, $\sigma_{t}$ is the standard deviation of the target, $\sigma_{b}$ is the standard deviation of the background. $C_{1}$, $C_{2}$ and $C_{3}$ are constants.

According to the above evaluation metrics, we calculated the simulated data of frozen lakes scene at different altitudes (10–100 km). The data are divided into two parts, the area including the flame by overlapping (the upper parts of Table 1) and without flame (the bottom of Table 1). The results are shown in Table 1.

## 4. Simulation Results

According to the analysis results of simulation experiment, three indicators: En, Var and SCR can reflect the infrared radiation response best. By using these three indicators, the differences (intensity in imagery) between background and target are well represented, that they indicate Information richness, texture characteristics and radiation intensity, respectively. Thus, these indicators are chosen to evaluate the detectability of the plume. The detectability of the infrared radiation of the plume at different flight altitudes is investigated below. The calculation area is a rectangular area of 61×21 size centered on the jet target. The size of the calculation window depends on the actual size of the simulation target.

**Figure 5:**
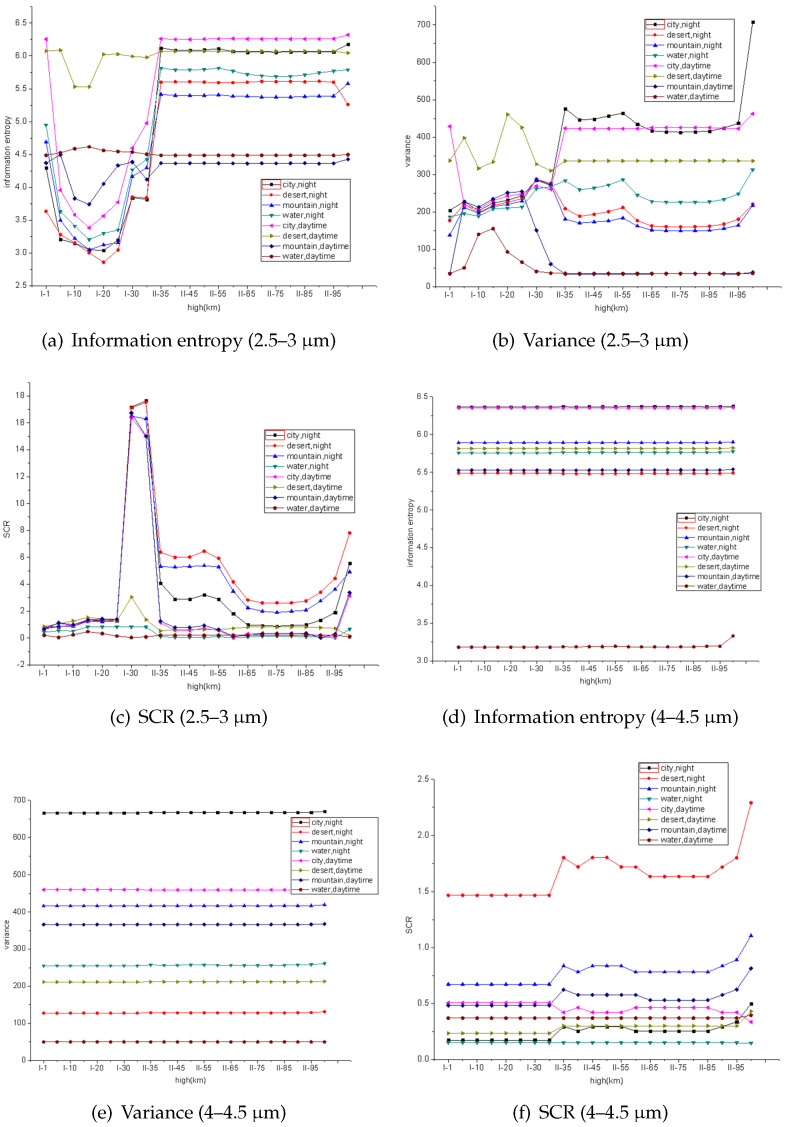
Detectability evaluation results at different wave bands.

From Figure 5, we can see that the three curves in 2.5–3 μm band (a,b,c) have obvious variation regularity, which indicates that the infrared radiation of the flame and background can be distinguished obviously. However, the curves in 4–4.5 μm band (d,e,f) is basically flat and excessive. The difference and discrimination of radiation indices are very small.

## 5. Conclusions and Discussion

The calculation models for infrared radiation of the earth background and missile plume are constructed in this paper. The image quality of the simulation data, that are formed by overlapping the plume on the background, are evaluated to study the detectability of the plume radiation. Based on the above analysis and investigation, we summarize as follows:

(1) In the detection period, the observation condition at night is better than that at daytime. The difference is mainly reflected in the observation of level II targets (second-stage engine exhaust with the same propellant), which can be clearly observed at night at all heights but it is difficult to observe the level II targets at all heights during the day. On the one hand, the infrared radiation of level II engines is less than that of level I engines, on the other hand, the background radiation in the day is greater than that in the night.

(2) We can see from the simulation results, three indicators response, such as entropy, SNR and variance, is relatively sensitive in 2.5–3 μm detector band.

(3) In terms of altitude, the visibility of the target at a higher altitude is higher. The infrared radiation of the engine can be clearly observed at or above 10 km in both day and night conditions. The reason is that the attenuation effect of the atmosphere above 10 km on the plume radiation is reduced.

The above conclusions are drawn from the selected typical surface scenes and cloudless conditions. In the actual flight process, the engine is located in a complex background and meteorological conditions, which require specific analysis and calculation according to the specific detection conditions.

## Figures and Tables

**Table 1:** Detectability results of different indicators on the lake scene.

	Indicator	PSNR	Contrast	SSIM	En	Hom	Smo	Var	Skew	SCR	Kur
Altitude											
Including Flame	10 km	43.32	36.08	0.97	4.70	7.2 × $10^{-2}$	0.40	210.20	5.9 × $10^{3}$	0.55	2.7 × $10^{5}$
	20 km	40.04	38.00	0.95	4.71	7.0 × $10^{-2}$	0.41	214.60	5.9 × $10^{3}$	1.23	2.7 × $10^{5}$
	30 km	39.99	64.09	0.93	4.96	6.1 × $10^{-2}$	0.63	330.90	1.1 × $10^{4}$	1.91	6.1 × $10^{5}$
	40 km	38.39	68.41	0.91	5.06	5.4 × $10^{-2}$	0.65	345.10	1.1 × $10^{4}$	2.14	6.2 × $10^{5}$
	50 km	37.91	94.92	0.88	5.08	5.5 × $10^{-2}$	0.71	399.80	1.4 × $10^{4}$	3.59	8.6 × $10^{5}$
	60 km	36.19	150.77	0.84	5.28	4.7 × $10^{-2}$	0.85	602.10	3.2 × $10^{4}$	5.54	2.8 × $10^{6}$
	70 km	35.63	167.29	0.83	5.33	4.4 × $10^{-2}$	0.88	704.30	4.3 × $10^{4}$	6.38	4.2 × $10^{6}$
	80 km	34.86	239.91	0.81	5.53	3.6 × $10^{-2}$	0.93	962.40	8.1 × $10^{4}$	8.11	1.0 × $10^{7}$
	90 km	34.96	224.68	0.81	5.52	3.8 × $10^{-2}$	0.93	900.70	7.0 × $10^{4}$	7.61	8.4 × $10^{6}$
	100 km	33.01	213.19	0.77	5.66	3.1 × $10^{-2}$	0.96	1.2 × $10^{3}$	1.1 × $10^{5}$	10.21	1.4 × $10^{7}$
Without Flame	10 km	38.11	43.95	0.93	5.56	2.6 × $10^{-2}$	0.29	161.90	−1.9 ×$10^{3}$	1.74	1.2 × $10^{5}$
	20 km	37.54	46.07	0.92	5.61	2.6 × $10^{-2}$	0.31	171.20	−1.7 ×$10^{3}$	2.46	1.3 × $10^{5}$
	30 km	37.80	56.53	0.90	5.63	2.6 × $10^{-2}$	0.35	188.30	−841.50	3.74	1.8 × $10^{5}$
	40 km	36.99	60.16	0.89	5.66	2.5 × $10^{-2}$	0.41	211.70	290.90	4.14	2.5 × $10^{5}$
	50 km	36.54	87.18	0.87	5.70	2.4 × $10^{-2}$	0.56	288.60	6.7 × $10^{3}$	6.47	8.4 × $10^{5}$
	60 km	35.36	119.96	0.84	5.74	2.4 × $10^{-2}$	0.78	476.30	2.4 × $10^{4}$	8.15	2.7 × $10^{6}$
	70 km	34.91	121.29	0.83	5.79	2.3 × $10^{-2}$	0.81	533.50	2.8 × $10^{4}$	8.07	3.0 × $10^{6}$
	80 km	34.03	140.35	0.82	5.83	2.2 × $10^{-2}$	0.85	600.80	3.4 × $10^{4}$	7.99	3.7 × $10^{6}$
	90 km	34.18	139.90	0.82	5.83	2.2 × $10^{-2}$	0.85	597.80	3.3 × $10^{4}$	8.15	3.6 × $10^{6}$
	100 km	32.53	153.77	0.79	5.75	2.4 × $10^{-2}$	0.92	856.80	6.4 × $10^{4}$	11.19	7.1 × $10^{6}$
